# Psychometric analysis of the questionnaires for the assessment of upper limbs available in their Italian version: a systematic review of the structural and psychometric characteristics

**DOI:** 10.1186/s12955-021-01891-w

**Published:** 2021-11-22

**Authors:** Luca Barni, María Ruiz-Muñoz, Manuel Gonzalez-Sanchez, Antonio I. Cuesta-Vargas, Jose Merchan-Baeza, Marco Freddolini

**Affiliations:** 1 Terme Redi, Montecatini Terme, Italy; 2 Department of Nursing and Podiatry, Faculty of Health Sciences, University of Málaga, Arquitecto Francisco Peñalosa, 3, 29071 Málaga, Spain; 3 Institute of Biomedicine of Málaga (IBIMA), 29010 Málaga, Spain; 4 Department of Physiotherapy, Faculty of Health Sciences, University of Málaga, 29071 Málaga, Spain; 5 School of Clinical Sciences of the Faculty of Health, Queensland University of Technology, Brisbane, QLD 4000 Australia; 6 Grupo de investigación Methodlogy, Methods, Models and Outcomes of Health and Social Sciences (M30), Facultad de Ciencias de la Salud y Bienestar, Universidad de Vic-Universidad Central de Cataluña (UVIC-UCC), Vic, Barcelona, Spain; 7 University of Florence, Firenze, Italy

**Keywords:** Upper Limb, Review, Psychometrics, Questionnaires, Pathology

## Abstract

**Introduction:**

There is no systematic review that analyzes the psychometric properties of questionnaires in Italian. Previous studies have analyzed the psychometric characteristics of instruments for the measurement of pathologies of upper limbs and their joints in different languages. The aim of the present study was to analyze the psychometric properties of the questionnaires published in Italian for the evaluation of the entire upper limb or some of its specific regions and related dysfunctions.

**Evidence acquisition:**

For the development of this systematic review, the following databases were used: PubMed, Scopus, Cochrane, Dialnet, Cinahl, Embase and PEDro. The selection criteria used in this study were: studies of transcultural adaptation to Italian of questionnaires oriented to the evaluation of upper limbs or any of their structures (specifically shoulder, elbow and wrist/hand), and contribution of psychometric variables of the questionnaire in its Italian version.

**Evidence synthesis:**

After reading the titles and applying the inclusion and exclusion criteria to the complete documents, 16 documents were selected: 3 for the upper limb, 8 for the shoulder, 1 for the elbow and 4 for the wrist and hand. The cross-sectional psychometric variables show levels between good and excellent in all the questionnaires. Longitudinal psychometric variables had not been calculated in the vast majority of the analyzed questionnaires.

**Conclusions:**

Italian versions of the questionnaires show good basic structural and psychometric characteristics for the evaluation of patients with musculoskeletal disorders of the upper limb and its joints (shoulder, elbow and wrist/hand).

## Introduction

Upper limb neuromusculoskeletal disorders are a common musculoskeletal complaint, with lifetime prevalence in developed nations of up to 67% [1]. It has been estimated that upper limb disorders cause at least 10% of the consultations of physiotherapists [2], generating a very high indirect health expense, due to their long recovery period, and thus leading to the loss of functional and working capacity [3].

In the clinical practice, there are objective and subjective instruments for the assessment and monitoring of these pathologies [4, 5]. Questionnaires are a necessary part of the process of managing patients' health. These tools are used to a large extent to objectively determine any response or change on the health status and function of the patient, with the latter reflecting his/her health status [4–6]. They help clinicians and researchers to monitor the situation of patients and determine whether they have changed [4–6]. As this form of patient-centered process management has been adopted and improved in the field of trauma over the past two decades, there has been an increase in the use of specific questionnaires for certain areas of the body. Consequently, they are frequently used as the standard protocol for the measurement and management of the functional status [6]. It is necessary that all measurement instruments used in the clinical practice and in research have been the subject of a validation study in which their psychometric characteristics are identified [7].

Italian is one of the official languages of the European Union, and it is spoken in eight countries [8]. Around the world, more than 65 million people speak Italian, becoming, recently, the fourth most studied language in the world [8].

Previous reviews have analyzed the psychometric characteristics of instruments for the measurement of pathologies of upper limbs and their joints in different languages [9–15]. In addition, a systematic review that analyzes the psychometric characteristics of the questionnaires in Italian for the cervical and lumbar spine has been published [16]. However, no systematic review has analyzed the psychometric characteristics of Italian tools for the assessment and follow-up of patients with upper limb disorders.

The aim of the present study was to analyze the psychometric properties of the questionnaires published in Italian for the evaluation of the entire upper limb or some of its specific regions and related dysfunctions.

## Methods and materials

### Protocol

This systematic review was carried out in accordance with the general guidelines and recommendations made by Preferred Reporting Items for Systematic Reviews and Meta-analyses (PRISMA) [7]. This systematic review was recorded at PROSPERO with the following reference number: CRD42020164002.

### Sources and search

The search was carried out in 7 databases, specifically: PubMed, Cochrane, PEDro, Cinahl, Scopus, Dialnet and Embase. The searches focused on the bibliographic review referring to the upper limbs or to any of the joints/segments included in this body region. A combination of the following keywords was carried out, using the Boolean operators “OR” and “AND”: upper limb, wrist, hand, elbow, shoulder, questionnaire, Italian, psychometric, validity and validation. No filter was used in the search.

### Eligibility criteria

The selection criteria used in this study were: studies of transcultural adaptation to Italian of questionnaires oriented to the evaluation of upper limbs or any of their structures (specifically shoulder, elbow and wrist/hand), and contribution of psychometric variables of the questionnaire in its Italian version. Articles that were published in languages other than English or Italian were excluded. Moreover, in the case of questionnaires with different updates, we selected the most recent version of the questionnaire validated in Italian. Articles published until November 30th, 2020, were considered.

### Study selection

After performing the bibliographic search, studies were first filtered based on the title and abstract. Subsequently, the selected documents were read in-depth in order to be included or excluded from the study.

The bibliographic search and study selection were carry out by two authors, who were mutually blinded in each of the different stages in which the search and selection of the studies was structured. In cases of discrepancy, a third author (with more than 15 years of experience in the identification and selection of scientific documents), decided whether the document was finally selected or not.

### Synthesis of results and data extraction

From each article, both the structural characteristics and the psychometric aspects of each of the questionnaires were extracted. The structural characteristics extracted were: full name, acronym, author and date of the adaptation to the Italian language, what it measures, number of items, completion time, result scale and cost. The psychometric aspects were: Standard Error of Measurement (SEM), Minimum Detectable Change (MDC), test–retest reliability, internal consistency, criterion validity, construct validity and sensitivity to changes. In addition, cross-cultural validity was assessed in each questionnaire [7].

## Results

### Search and selection of documents

**Fig. 1:**
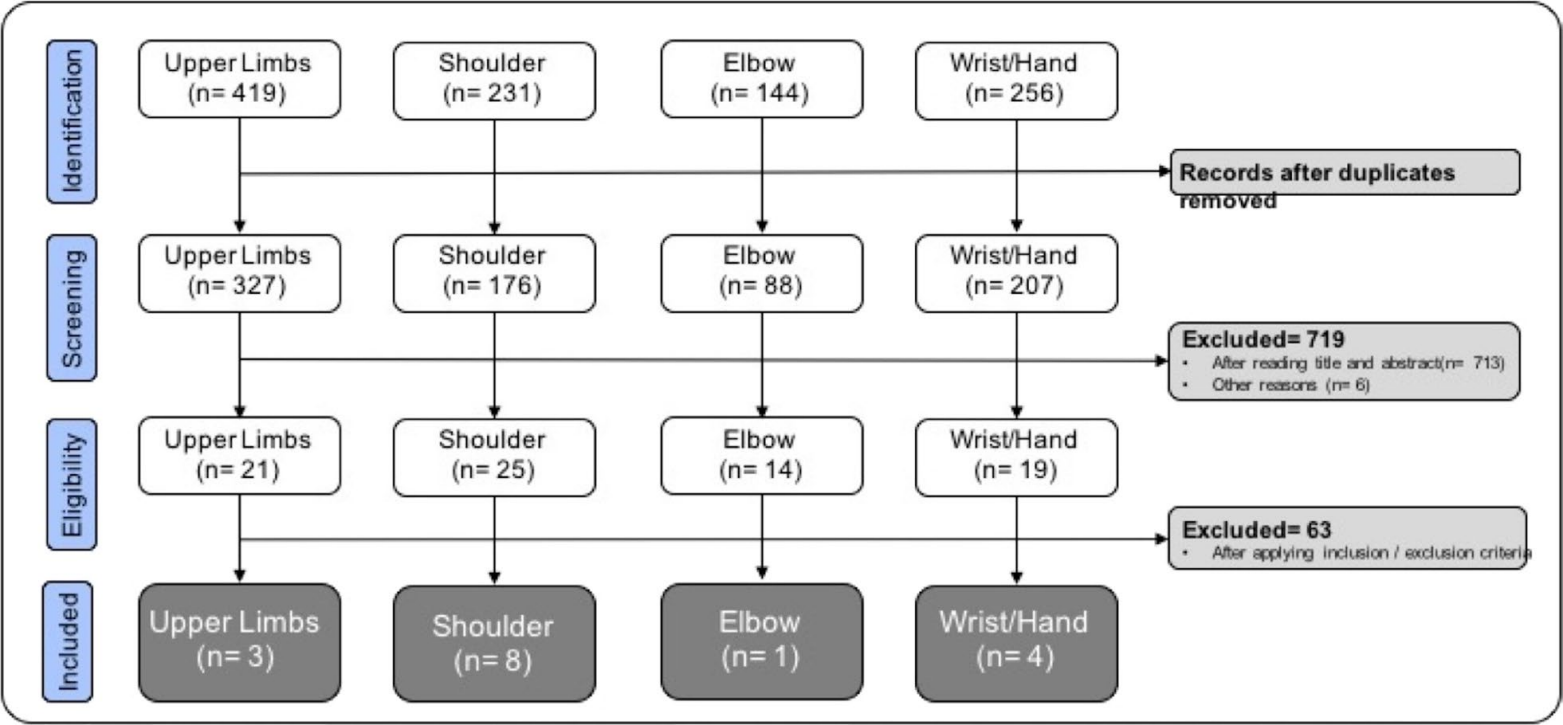
Flow chart of the search, selection and inclusion of the questionnaires for the evalution of the upper limb avaibale in Italian version

Figure 1 shows the flowchart of the search and selection papers. After the initial identification of 1050 studies and the removal of duplicates, 798 documents were selected. These were classified in the following manner: 327 documents for the upper limbs, 176 for the shoulder, 88 for the elbow and 207 for the wrist and hand. After reading the titles and applying the inclusion and exclusion criteria to the complete documents, 16 documents were selected.

These included three for the upper limb (the Disability of the Arm, Shoulder and Hand [17], the Cold Intolerance Symptom Severity questionnaire [18] and the Upper Limb Functional Index [19]), eight for the shoulder, one for the elbow and four for the wrist and hand (see Fig. 1 for more details).

### Structural characteristics

From a structural point of view, Tables 1, 2, 3 and 4 present the structural characteristics of the questionnaires for the upper limbs, shoulder, elbow and hand/wrist, respectively. For the upper limbs in particular (Table 1), the number of items of the identified questionnaires ranged from 14 to 30, the number of sub-categories ranged from 0 to 7, the time to complete the questionnaires ranged from 5 to 10 min and all the questionnaires were free.

**Table 1:** Structural characteristics of the questionnaires identified for the upper limb

Name	Acronym	No of items	Sub-category	Time to complete	Measurement	Cost
Disability of the Arm, Shoulder and Hand [7]	DASH	30	DASH function/symptoms DASH sport/music DASH work	10 min	0–100	Free
Cold Intolerance Symptom Severity questionnaire [17]	CISSq	14	Pain Numbness Stiffness Weakness Aching Swelling Skin colour change	10	0–100	Free
Upper Limb Functional Index [18]	ULFI	25	-	5	0–100	Free

**Table 2:** Structural characteristics of the questionnaires identified for the shoulder

Name	Acronym	No of items	Sub-category	Time to complete	Measurement	Cost
American Shoulder and Elbow Surgeons [24]	ASES	11	Pain Function	5 min	100	Free
Kerlan–Jobe Orthopaedic Clinic Shoulder and Elbow [21]	KJOCSE	10		10 min	100	Free
Nottingham Clavicle Score [19]	NCS	10	Pain in bed at night Ability to lift heavy objects and overhead strength Cosmetic satisfaction Movements and clicking within the shoulder Ingling and numbness in the arm and neck Heavy or dragging sensations in the arm	10 min	100	Free
Oxford Shoulder Score [20]	OSS	12	–	3–5 min	12–60	Free
Rotator Cuff Quality of Life [25]	RC-QoL	30	Physical alterations Job Recreational/sports activities Social aspects Lifestyle	10–15 min	0–300	Free
Simple Shoulder Test [26]	SST	12	–	5 min	0–100	Free
Shoulder Pain and Disability Index [27]	SPADI	13	Pain Function	5 min	0–100	Free
University of California—Los Angeles [26]	UCLA	2	Pain Function	3 min	0–10 0–10	Free
Western Ontario Osteoarthritis of the Shoulder [22]	WOOS	19	Physical symptoms Sport/recreation/work Lifestyle Emotions	10 min	0–1900	Free
Western Ontario Shoulder Instability [23]	WOSI	21	Physical symptoms Sport/recreation/work function Lifestyle function Emotional well-being	10 min	0–2100	Free

**Table 3:** Structural characteristics of the questionnaires identified for the elbow

Name	Acronym	No of items	Sub-category	Time to complete	Measurement	Cost
Patient-rated tennis elbow evaluation [28]	PRTEE	15	Pain Degree of difficulty (specific activities) Degree of difficulty (usual)	10	0 (best)–100 (worst)	Free

**Table 4:** Structural characteristics of the questionnaires identified for the wrist

Name	Acronym	No of items	Sub-category	Time to complete	Measurement	Cost
Functional index for hand osteoarthritis [29]	FIHOA	10	–	5 min	0–30	Free
Hands Mobility in Scleroderma [30]	HAMIS	9	–	15 min	0 (best)–27 (worst)	Free
Hand functional disability scale [31]	HFDS	18	Hand ability in: The kitchen Dressing Personal hygiene Office tasks Other general items	10 min	0–90	Free
Patient-rated wrist/hand evaluation [32]	PRWHE	15	Pain Function	10 min	0–100	Free

For their part, the questionnaires identified for the specific assessment of the shoulder had a number of items that ranged from 2 to 30, the number of subcategories ranged from zero to six, the time to complete them ranged from 3 to 15 min, and all the questionnaires can be used free of charge (Table 2). Regarding the evaluation of the elbow, a single free questionnaire was identified, which had 15 items and 3 sub-scales and requires approximately 10 min to complete (Table 3).

Finally, Table 4 shows the questionnaires for the hand and the wrist. The number of items in these questionnaires ranged from 9 to 18, the sub-categories ranged from 0 to 5 and the time to complete them ranged from 5 to 15 min. All the identified questionnaires can be used free of charge.

### Psychometric characteristics

The psychometric characteristics of the questionnaires are presented in Tables 5, 6, 7 and 8 for the upper limbs, shoulder, elbow and hand/wrist, respectively. Specifically, Table 5 shows the psychometric characteristics of the questionnaires for the upper limbs. It presents the reliability of all the questionnaires, ranging from good to excellent. Similarly, the internal consistency was excellent for all the questionnaires, while two questionnaires (CISS and ULFI) [18, 19] perform an analysis of error measures and one questionnaire (DASH) analyzes the difficulty of the response [17].

**Table 5:** Psychometric variables of the selected questionnaires for the upper limb

Name	Reliability test-restest	Inter-observer reliability	Internal consistency	Validity of criteria	Sensitivity	SEM	MDC	Responsiveness (SRM—standardised response mean)
Disability of the Arm, Shoulder and Hand [7]	DASH-FS: 0.89 DASH-SM: 0.75 DASH-W: 0.84	–	DASH-FS: 0.90 DASH-SM: 0.82 DASH-W: 0.85	DASH-FS: 0.27–0.70 DASH-SM: 0.01–0.5 DASH-W: 0.36–0.63	–	–	–	0.90
Cold Intolerance Symptom Severity questionnaire [17]	CISS: 0.96	CISS: 0.34–0.92	CISS total: 0.93 CISS scales: 0.58–0.91	DN4: 0.73 MRC: 0.44–0.61	–	4.07 points	MDC90: 9.45 MDC95: 11.30	–
Upper Limb Functional Index [18]	ULFI: 0.94	–	ULFI: 0.90	DASH:0 81	–	5 points	MDC90: 12	–

**Table 6:** Psychometric variables of the selected questionnaires for the shoulder

Name	Reliability test-restest	Inter-observer reliability	Internal consistency	Validity of criteria	Sensitivity	SEM	MDC	Responsiveness (SRM—standardised response mean)
American Shoulder and Elbow Surgeons [24]	0.91	–	0.85	OSQ: 0.78 DASH: − 0.92 SF-36: 0.20–0.60	–	–	–	–
Kerlan–Jobe Orthopaedic Clinic Shoulder and Elbow [21]	0.99	–	0.910	DASH: − 0.697	–	0.81	2.42	–
Nottingham Clavicle Score [19]	0.29–0.90	–	0.86	OSS: − 0.84 DASH: − 0.87 SF-36: 0.19–0.74	–	–	–	–
Oxford Shoulder Score [20]	0.57–0.82	0.97	0.95	UCLA: 0.67 Constant-Murley: 0.73 SF-36: 0.40–0.74	–	–	–	–
Rotator Cuff Quality of Life [25]	0.94	–	0.95	–	–	–	–	–
University of California—Los Angeles [26]	> 0.89	–	–	SPADI: > 0.91 SST: > 0.91	–	–	–	–
Shoulder Pain and Disability Index [27]	> 0.89	–	–	UCLA: > 0.91 SST: > 0.91	–	–	–	–
Simple Shoulder Test [26]	> 0.89	–	–	SPADI: > 0.91 UCLA: > 0.91	–	–	–	–
Western Ontario Osteoarthritis of the Shoulder [22]	0.96	–	0.910	DASH: 0.73	–	0.80	2.22	Physical symptoms: 0.98 Sport/recreation/work: 1.30 Lifestyle: 1.13 Emotions: 0.81 Total:1.11
Western Ontario Shoulder Instability [23]	0.95–Short term 0.93-Medium term	–	0.93	DASH: 0.79 SF-36: 0.11	–	0.71	1.96	WOSI: 1.57

**Table 7:** Psychometric variables of the selected questionnaires for the elbow

Name	Reliability test-restest	Inter-observer reliability	Internal consistency	Validity of criteria	Sensitivity/specificity	SEM	MDC	Responsiveness
Patient-Rated Tennis Elbow Evaluation [28]	0.95 short term 0.93 long-term	–	0.95	DASH Overall Score: 0.84 Pain: 0.77 Functional Ability: 0.79	Sensitivity: 0.94 Specificity: 0.78	2.68 short term 3.25 long-term	–	ES: 2.0 SRM: 2.3

**Table 8:** Psychometric variables of the selected questionnaires for the wrist

Name	Reliability test-restest	Inter-observer reliability	Internal consistency	Validity of criteria	SEM	MDC	Responsiveness (SRM—standardised response mean)
Functional index for hand osteoarthritis [29]	0.955 (0.767–0.979)	–	0.87	VAS—pain: 0.488 HAQ: 0.609 SF-36: (-0.283) – (-0.637)	–	–	–
Hands Mobility in Scleroderma [30]	0.99 (both hands)	–	0.94 right hand 0.93 left hand	SF-36—PSI: − 0.36 SF-36—MSI: − 0.36	–	–	–
Hand functional disability scale [31]	0.96 (0.83–0.97)	–	0.872 (0.637–0.928)	HAQ: 0.81	–	–	–
Patient-rated wrist/hand evaluation [32]	–	–	0.96	DASH: 0.80–0.81 SF-36: (− 0.41)–(− 0.47)	–	–	–

Table 6 shows the results of the psychometric characteristics of the questionnaires aimed at evaluating the shoulder. Specifically, 8 of the 10 selected questionnaires present excellent reliability, which is between poor and excellent for NCS [20] and between moderate and excellent for the Oxford Shoulder Score [21]. The internal consistency presents excellent results in 7 of the 10 analyzed questionnaires, while in the other 3 questionnaires this psychometric variable is not shown. To analyze the criterion validity, the SF-36 and DASH questionnaires have been frequently used, each of them in four validation studies. In 3 of the 10 studies, error measures (SEM and MDC) were analyzed, specifically in KJOCSE [22], WOOS [23] and WOSI [24]. In addition, in the latter two, criteria of responsiveness (SRM—standardized response mean) were also analyzed, being the only two that assessed this psychometric characteristic among all the identified questionnaires for the evaluation of the shoulder.

Table 7 presents the psychometric characteristics of the only questionnaire identified in Italian for the evaluation of the elbow. Specifically, the reliability of this questionnaire is 0.95 in the short term, and 0.93 in the long term. In addition, the internal consistency is 0.90 and the DASH questionnaire was used, again, for criterion validity. This questionnaire is one of the few that perform an analysis of sensitivity and specificity. The sensitivity is 0.94 while the specificity is 0.78. In addition, the SEM was analyzed both in the short and long term, as well as the ease of response, analyzing both the ES and the SRM.

Table 8 presents the psychometric characteristics of the questionnaires in Italian aimed at evaluating the hand and the wrist. The test–retest reliability of these standardized questionnaires was calculated in three of the four selected documents, showing values above 0.95 in all cases. On the other hand, although the internal consistency is excellent, it decreases slightly, showing values between 0.87 and 0.96. For criterion validity, again, the SF-36 questionnaire is the most used, since it was used in three of the four selected questionnaires. None of them analyze sensitivity, error measurement or responsiveness.

### Cross-cultural validity

Table 9 presents the analysis of the cross-cultural validity for all the questionnaires included in the study, following the different items present in the COSMIN guide for the evaluation of this characteristic [7]. It can be seen how all the selected questionnaires carry out a cross-cultural translation process following the recommendations of the literature, however, in the cross-cultural population process, how none of them performed a confirmatory factor analysis, only one presented an adequate one. sample coma and 11 of them carried out a pre-evaluation of the questionnaire before being used with a larger sample. For more details on the cross-cultural validation evaluation, see Table 9.

**Table 9:** Evaluation of the cross-cultural validity of all the questionnaires included in the present study, considering the criteria of the COSMIN guide

	% missing items	Descripiton missing items	Adequate sample size	Translation description	Translated by experts	Independent Translators	Forward backward translation	Original/ translation differences solved	Translation reviewed	HR-PRO pretested	Pretested sample describe	Samples similar	Important flaw	CFA performed	DIF assessed
CISS	-	-	-	•	•	•	•	•	•	•	•	-	•	-	-
DASH	-	-	-	•	•	•	•	•	•	•	•	-	•	-	-
ULFI	-	-	-	•	•	•	•	•	•	•	-	-	-	-	-
ASES	-	-	-	•	•	•	•	•	•	-	-	-	•	-	-
KJOCSG	•	-	-	•	•	•	•	•	•	•	-	-	•	-	-
NCS	•	•	-	•	•	•	•	•	•	•	•	•	•	-	-
OSS	-	-	•	•	•	•	•	•	•	•	•	-	•	-	-
RC-QOL	-	-	-	•	•	•	•	•	•	-	-	-	-	-	-
UCLA	•	-	-	•	•	•	•	•	•	-	-	-	-	-	-
SPADI	•	-	-	•	•	•	•	•	•	-	-	-	-	-	-
SST	•	-	-	•	•	•	•	•	•	-	-	-	-	-	-
WOOS	-	-	-	•	•	•	•	•	•	-	-	-	-	-	-
WOSI	•	-	-	•	•	•	•	•	•	•	•	•	•	-	-
PRTEE	•	-	-	•	•	•	•	•	•	•	•	-	•	-	-
FIHOA	-	-	-	•	•	•	•	•	•	-	-	-	-	-	-
HAMIS	-	-	-	•	•	•	•	•	•	•	-	-	-	-	-
HFDS	-	-	-	•	•	•	•	•	•	•	•	-	-	-	-
PRWHE	-	-	-	•	•	•	•	•	•	•	•	-	•	-	-

## Discussion

The objective of the present study was to gather all the existing questionnaires available in Italian for the assessment of the upper limb, both generally and for each of its main joints (shoulder, elbow and hand/wrist), in order to compile both the structural and psychometric characteristics of all the questionnaires, as well as to compare them, with the aim of identifying the most interesting questionnaire, based on its clinical and research use. The psychometric characteristics of the identified questionnaires generally show good or very good reliability and internal validity values. The construct validity depends on the variable to be analyzed and it is observed that there are variables with a very good correlation while others show a poor correlation (Tables 5, 6, 7 and 8). These results are similar to those observed in a previous study where the psychometric characteristics of the questionnaires published in Italian were analyzed, although for the evaluation of the lumbar and cervical spine [16].

In the scientific literature, most of the questionnaires developed to test pain, function and social influence are in English [17, 24]. They are frequently used in clinical and research fields in the Anglo-Saxon culture, and their demand is increasingly spreading all over the world [34, 35]. This leads to the problem of cultural and linguistic differences between various countries, which can pose difficulties in terms of the equivalence of translated questionnaires to the original versions [17, 24]. Therefore, validation in the desired language must comply with standards that are as homogeneous and rigorous as possible in the scientific literature [24, 35]. The validation process must allow the different versions to be made and developed in different parts of the world, to be culturally and linguistically adapted, and to be comparable amongst themselves in order to use them for higher evaluations, such as reviews and meta-analyses [6, 36, 37].

### Selection and use of questionnaires in a clinical and research environment

A total of 16 questionnaires were identified and validated in Italian for the assessment of the upper limbs, shoulder, elbow and wrist/hand. Each of these questionnaires has a series of different psychometric characteristics, as well as a different outcome variable. Therefore, it will be the clinician or researcher, depending on the outcome variable of interest, who decides which of these questionnaires best suits the needs or objectives, based on the available time, patient profile, main variable of interest, etc.

In the selection of the questionnaires, from the clinical utility point of view, there are usually two characteristics that exert a stronger influence when they are selected: time to complete the questionnaire and main outcome variable.

For the upper limb, DASH and CISSq take about 10 min to complete. The ULFI questionnaire, as in the case of DASH, measures upper limb function in people with musculoskeletal impairment, although it takes less time to be completed (5 min), while CISSq assesses the severity of cold intolerance in a patient population with surgical repair of peripheral nerve lesions in the upper limb.

In the questionnaires aimed at evaluating the shoulders, there is enormous heterogeneity when defining the objective or the main outcome variable of the questionnaire, each of them being very specific for a specific variable. However, in the time to complete it, there is an almost generalized homogeneity, ranging between 5 and 10 min, with the exception of the UCLA Shoulder Score, which is the one that requires the shortest time to be completed and the Rotator Cuff Quality of Life, which is, with 10–15 min, the identified questionnaire that requires the longest time to complete (Table 2).

For the elbow, only the PRTEE was selected. It takes about 10 min to be completed and is designed to evaluate pain and disability in subjects with lateral elbow tendinopathy.

In the questionnaires aimed at evaluating the wrist, there is also a lot of heterogeneity in the main outcome variables that each questionnaire assesses, although all of them take at least 10 min to complete, with the exception of FIHOA [30], which requires 5 min (Table 4).

On the other hand, it is important to mention that, during the bibliographic search, it was identified that different questionnaires are aimed at fully or partially evaluating some of these regions, but whose Italian version had not been validated yet. In this sense, it would be interesting for future studies to develop Italian versions and expand the catalogue of available tools for these body regions.

### Psychometric characteristics of questionnaires for upper limbs

In general, both the reliability and internal consistency of the questionnaires identified for the evaluation of the upper limbs is similar to that of their respective original versions, i.e., CISS [40], DASH [41] and ULFI [46], being comparable with other versions published in other languages, such as: the Swedish [38] and Turkish versions [39] for the CISS; Swedish [42], Danish [43] and Dutch versions [44] in the case of DASH; and Spanish [47], French [50] and Korean versions [48] for the ULFI.

Regarding the criterion validity, each questionnaire uses different reference tools. The validity of DASH is similar to that of the original English version [51] and Swedish translation [42], which uses the SF-36 [45]. The DASH questionnaire is the validation instrument for the ULFI questionnaire, whose results are in line with those of the English [46] and French versions [49] and higher than those of the Korean version [48]. Only the Spanish version uses the EQ-5D-3 [47]. For the CISS questionnaire, validity analyses were not performed for the English version [40].

The SEM value is only reported for the CISS [18] and ULFI [51] questionnaires. This parameter is reported by other studies only for ULFI. Specifically, the value is slightly higher than that of the English [46, 52] and Spanish versions [47] and lower than that of the French version [49].

The MDC value is only reported for the ULFI [51] and CISS [18] questionnaires. The MDC value reported in the English ULFI [46, 52] is lower than that reported in the Italian version [51].

### Psychometric variables of the questionnaires for the assessment of the shoulder

All the questionnaires aimed at evaluating the shoulder show excellent reliability, with the exception of the Nottingham Clavicle Score [20] and the Oxford Shoulder Score [21], which present two sub-scales with poor (0.29, NCS) and moderate (0.57, OSS) reliability levels (Table 6). When compared with the original version, some questionnaires are coherent with their Italian versions, such as the Italian UCLA scale [65, 66], KJOCSE [22], RC-QoL [26], ASES [25], WOOS [23] and WOSI [24]. However, reliability is lower than that of the original version, as is the case of SPADI [65, 67, 68]. Except for specific exceptions, all these questionnaires are also in line with other versions published in other languages, with population groups as diverse as Chinese [64], Turkish [57, 63], Korean [56, 59], Polish [60], Hebrew [77], French [61], Persian [24, 62], German [54, 58], Finnish [55] and Spanish [28, 53], among others. The fact that there are multiple versions of the same questionnaire and that, in addition, they have similar psychometric characteristics, makes it possible to compare the results of different studies, thus expanding the possibility of understanding the eventual compared results.

The same trend regarding internal consistency was observed when comparing the Italian questionnaires with the different original versions. In this sense, they are all consistent with the levels observed in the original version, as well as with different versions made in other languages, although there are specific exceptions, such as SPADI, which presents a slightly lower level with respect to that of the Dutch, German, Greek and Slovene versions [24, 69–71], and SST [65], which presents values higher than that of the Spanish and Dutch versions [69, 70].

For external validity, there is considerable consensus when it comes to selecting reference tools to calculate this variable. Specifically, there are 4 instruments that are used on a recurring basis. The SF-36 is used by ASES [25], NCS [20], OSS [21] and WOSI [24], DASH is used by KJOCSE [22], NCS [20], WOOS [23] and WOSI [24], and UCLA is used by OSS [21], SPADI [28] and SST [27].

The levels of correlation observed in the Italian versions of the questionnaires are in line with those of other versions of the selected questionnaires. In fact, the SF-36 is the questionnaire with the worst correlations with all the analyzed versions. However, in a generalized manner, the questionnaires aimed at evaluating the upper limbs in a specific way (DASH, UCLA, OSS) correlate much better both in the Italian versions and in the rest of the other versions, identifying values that range between moderate r ≥ 0.6 and excellent ≥ 0.9.

Most of the Italian versions of the selected questionnaires did not calculate SEM or MDC, except KJOCSE, WOOS and WOSI, with SEM values of 0.81, 0.80 and 0.71, respectively (Table 6) and MDC values of 2.42, 2.22 and 1.96, respectively (Table 6). However, it was calculated in other versions, such as in the Turkish [63] and Chinese [64] versions of RC-QoL, the Chinese [67], Greek [24] and English versions [71] of SPADI, and the Dutch version of SST [69]. This psychometric variable, in a generalized manner, is not calculated by the different versions of the selected questionnaires, except for the German version of ASES [54], the Spanish version of OSS and WOSI.

### Psychometric variables of questionnaires for the assessment of the elbow and comparison with other translations

Regarding the evaluation of the elbow, only the PRTEE questionnaire was found to have an Italian version.

Reliability was similar between the Italian version and the other analyzed versions [29, 72–75]; the value of the French version [76] is slightly lower.

Internal consistency was not reported for the Italian version [29], while all the other versions showed similar values between them [72–76].

Construct validity was assessed using correlation with DASH in all analyzed versions [72–76], whose values are similar to that of the Italian version [29].

The SEM value is reported with similar results in all analyzed versions [31, 72–74, 76], except in the Turkish [75] version, where it is not presented. Finally, the MDC value is reported with similar results only in the Dutch and French versions [72, 76].

### Psychometric characteristics of questionnaires for the hand and wrist and comparison with other translations

Regarding reliability, the value of the Italian FIHOA [30] is similar to that of the Dutch and Persian versions [72, 73], while the Korean [74] and Japanese [75] versions showed slightly lower values. The reliability of the Italian HAMIS [76] is similar to that of the Brazilian version [77], while only the original English version was found for the HFDS [78] with a value similar to that of the Italian version [32]. The value of the Italian PRWHE [33] is similar to that of the Arabic, Dutch and Turkish versions [79–81], and higher than that of the Hindi version [82].

Regarding internal consistency, the value of the Italian FIHOA [30] is similar to that of all the analyzed versions [72–75]. The Italian HAMIS [76] has a value similar to that of the Brazilian version [77]. For the HFDS, the internal consistency was analyzed only for the Italian version [32]. The value of the Italian PRWHE [33] is similar to that of the Arabic and Dutch versions [79, 80] and higher than that of the Hindi and Turkish versions [81, 82].

Regarding construct validity, all FIHOA questionnaire versions [30, 72–75] use the SF-36 questionnaire and/or VAS, as in the case of the Italian version. The value of the Italian HAMIS [76] is similar to that of the Brazilian version [77]. For HFDS, the Italian [32] and English [78] versions used the Health Assessment Questionnaire (HAQ) to evaluate validity, showing similar results. The validity of the Italian PRWHE [33] was evaluated using the correlation with SF-36 and DASH, as in the case of the other analyzed versions [79–81], except for the Hindi version [82], with similar results.

Regarding SEM, for the FIHOA questionnaire, only the Persian version [73] was evaluated, obtaining a value of 2. Neither the Italian nor the Brazilian version [76, 77] of HAMIS reports the SEM data. No version of HFDS reports the SEM value [32, 78]. PRWHE SEM data are reported only in the Hindi and Arabic versions [79, 82], with 5.4 and 3.7, respectively.

Regarding MDC, none of the analyzed FIHOA versions report the MDC value [30, 72–75]; only the Persian version [73] reports SDC, with a value of 5.4. Neither the Italian nor the Brazilian version [76, 77] of HAMIS reports the MDC value. No version of HFDS reports the MDC value [32, 78]. PRWHE MDC data are reported only in the Hindi and Arabic versions [79, 82], with 12.5 and 10.2, respectively.

It is important to note that some limitations were observed in the analyzed tools. Many of them do not have important psychometric variables such as sensitivity and error measurements. Therefore, future studies should be designed to analyze these psychometric variables, which are of great importance in research, especially in the clinical practice. Moreover, it is very important to consider that Italian is a language spoken by more than 65 million people living in at least 8 different countries. Thus, it is essential to consider the cultural characteristics of each population group that could condition the interpretation of both the questions and the answers obtained. In this sense, if the socio-demographic and cultural differences are substantial, it would be necessary to develop a specific version, completely adapted to the population group of interest.

On the other hand, it would be necessary to start introducing the clinimetric analysis of construct validity. Clinimetric analysis is a recently coined term that is defined as “*the science of clinical measurements*” [83], and allows the identification/creation of new variables/scales in traditional assessment tools. This new approach could provide very relevant clinical information, such as the fact that the items included in a scale may belong to an underlying clinical construct/dimension. You can also report on the degree of validity of the mean of a dimension that is being evaluated [84, 85]. Undoubtedly, this analysis would provide a greater understanding of the scale to be evaluated, also allowing a more accurate profile of the patient under evaluation based on the dimensions defined according to the analysis.

## Conclusions

The main conclusion that can be drawn from this study is that the Italian versions of the questionnaires show good basic structural and psychometric characteristics for the evaluation of patients with musculoskeletal disorders of the upper limb and its joints (shoulder, elbow and wrist/hand). Italian clinicians have different instruments with psychometric characteristics that, as a rule, resemble other versions of the same questionnaire published in other languages. Therefore, these characteristics would allow a comparison of the results obtained with samples from other countries. Despite these good features, there are psychometric variables that none of the selected questionnaires include. Thus, it is necessary to carry out studies that include psychometric variables in order to make the validation process homogeneous and identical for the scientific community.

## Data Availability

Not applicable.

## Abbreviations

ASES: American Shoulder and Elbow Surgeons
CISSq: Cold Intolerance Symptom Severity questionnaire
COSMIN: COnsensus-based Standards for the selection of health Measurement Instruments
DASH: Disability of the arm, shoulder and hand
DN-4: Douleur Neuropathique en 4 Questions
ES: Effect size
FIHOA: Functional index for hand osteoarthritis
HAMIS: Hands mobility in scleroderma
HAQ: Health assessment questionnaire
HFDS: Hand functional disability scale
KJOCSE: Kerlan–Jobe Orthopaedic Clinic Shoulder and Elbow
MDC: Minimum detectable change
MRC: Medical research council
NCS: Nottingham Clavicle score
OSQ: Oxford shoulder questionnaire
OSS: Oxford Shoulder Score;
PRISMA: Preferred Reporting Items for Systematic Reviews and Meta-analyses
PRTEE: Patient-Rated Tennis Elbow Evaluation
PRWHE: Patient-rated wrist/hand evaluation
RC-QoL: Rotator cuff quality of life
SEM: Standard error of measurement
SF-36: Short form health survey
SPADI: Shoulder pain and disability index
SRM: Standardized response mean
SST: Simple shoulder test
UCLA: University of California—Los Angeles
ULFI: Upper Limb Functional Index
VAS: Visual analogue scale
WOOS: Western Ontario Osteoarthritis of the Shoulder
WOSI: Western Ontario Shoulder Instability
