# Correction: Psychometric analysis of the questionnaires for the assessment of upper limbs available in their Italian version: a systematic review of the structural and psychometric characteristics

**DOI:** 10.1186/s12955-022-01982-2

**Published:** 2022-05-05

**Authors:** Luca Barni, María Ruiz‑Muñoz, Manuel Gonzalez‑Sanchez, Antonio I. Cuesta‑Vargas, Jose Merchan‑Baeza, Marco Freddolini

**Affiliations:** 1 Terme Redi, Montecatini Terme, Italy; 2 Department of Nursing and Podiatry, Faculty of Health Sciences, University of Málaga, Arquitecto Francisco Peñalosa, 3, 29071 Málaga, Spain; 3 Institute of Biomedicine of Málaga (IBIMA), 29010 Málaga, Spain; 4 Department of Physiotherapy, Faculty of Health Sciences, University of Málaga, 29071 Málaga, Spain; 5 School of Clinical Sciences of the Faculty of Health, Queensland University of Technology, Brisbane, QLD 4000 Australia; 6 Grupo de Investigación Methodlogy, Methods, Models and Outcomes of Health and Social Sciences (M30), Facultad de Ciencias de la Salud y Bienestar, Universidad de Vic-Universidad Central de Cataluña (UVICUCC), Vic, Barcelona, Spain; 7 Istituto Italiano di Tecnologia, Via Morego, 30, 16163 Genova, Italy

## Correction: Health and Quality of Life Outcomes  (2021) 19:259  https://doi.org/10.1186/s12955-021-01891-w

The original article [1] contained an incorrect institution for Affiliation #7 which has since been corrected.
